# Impact evaluation of malaria control interventions on morbidity and all-cause child mortality in Mali, 2000–2012

**DOI:** 10.1186/s12936-018-2573-1

**Published:** 2018-11-14

**Authors:** Kassoum Kayentao, Lia S. Florey, Jules Mihigo, Abdoul Doumbia, Aliou Diallo, Diakalia Koné, Ogobara Doumbo, Erin Eckert

**Affiliations:** 1 Malaria Research and Training Center (MRTC), University of Sciences, Techniques and Technologies of Bamako (USTTB), Bamako, Mali; 2 President’s Malaria Initiative (PMI), U.S. Agency for International Development (USAID), Washington, District of Columbia, USA; 3 US President’s Malaria Initiative, United States Agency for International Development (USAID), Bamako, Mali; 4 National Malaria Control Programme (NMCP), Bamako, Mali

**Keywords:** Malaria, Impact, Evaluation, Intervention, Under-five mortality, Mali

## Abstract

**Background:**

Major investments have been made since 2001, with intensification of malaria control interventions after 2006. Interventions included free distribution of insecticide**-**treated nets (ITN) to pregnant women and children under 5 years old, the introduction of artemisinin combination therapy (ACT) for malaria treatment, and indoor residual spraying of insecticides. Funders include the Government of Mali, the Global Fund to Fight AIDS, Tuberculosis and Malaria, and the US President’s Malaria Initiative.

**Methods:**

Data from nationally representative household surveys conducted from 2000 to 2015 was used to performed the trend analysis for malaria intervention coverage, prevalence of morbidities among children under 5 years old [parasitemia and severe anaemia (< 8 g/dl)], and all-cause mortality of children under 5 (ACCM). Prevalence of contextual factors likely to contribute to ACCM were also assessed. The impact of these interventions was assessed on malaria morbidity and mortality using a plausibility argument. With the assumption that malaria contributes significantly to under-five mortality in settings with high malaria transmission, associations between malaria control interventions and all-cause under-five mortality (ACCM) were assessed taking into account other contextual factors related to child survival.

**Results:**

Intervention coverage improved significantly from 2006 to 2012. Household ownership of ITN increased from 49% in 2006 to 84% in 2012. ITN use also increased over the same period, from 26% in 2006 to 69% in 2012 among children under 5 and from 28% in 2006 to 73% in 2012 among pregnant women. The coverage of intermittent preventive treatment in pregnancy (IPTp) using two or more doses of SP increased from 10% in 2006 to 29% in 2012. In 2010, 23% of febrile children under 5 received ACT, as opposed to 19% in 2012. The prevalence of *Plasmodium falciparum* infection increased from 2010 (38.6%) to 2012 (51.6%), followed by a decrease in 2015 (35.8%). The prevalence of severe anaemia decreased from 2010 (26.3%) to 2012 (20.6%) and continued to decline in 2015 (19.9%). An impressive decline in ACCM was observed, from 225 in 1997–2001 to 192 in 2002–2006 and 95 in 2008–2012. Changes in contextual factors such as climate, socio-economic, nutrition, and coverage of maternal and child health interventions over the evaluation period did not favour reductions in ACCM, and are therefore unlikely to explain the observed results.

**Conclusions:**

Taken as a whole, the evidence supports the conclusion that malaria control interventions substantially contributed to the observed decline in ACCM in Mali from 2000 to 2012, even in the context of continued high prevalence of parasitaemia explained by contextual factors such as climate change and political instability.

## Background

A long time before the discovery of the malaria parasite in 1880 by Alphonse Laveran [1], humanity had been burdened by malaria. Although the disease has been eradicated in many countries, in the majority of sub-Saharan African countries it is still a major public health problem, especially for children and pregnant women who are most at risk of severe disease and death [2], despite the intense global effort over the last two decades to defeat the disease. Most countries in sub-Saharan Africa are far away from the vision of a world free of malaria set by the global technical strategy for malaria 2016–2030 [3]. Nonetheless, countries where malaria impact evaluations have been conducted show a decrease in malaria morbidity and mortality following intensified investments in malaria prevention and control [4–7].

**Fig. 1:**
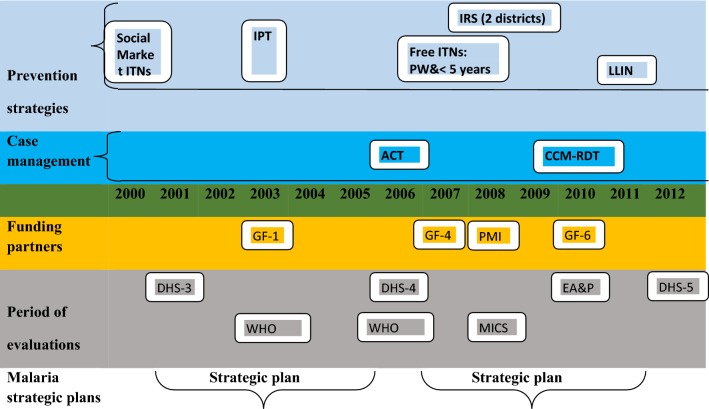
Timeline illustrating funding, evaluation periods and timing of implementation of malaria policies and interventions, Mali, 2000–2012. Between 2001 and 2012, the Government of Mali (GOM) and its international development partners invested heavily in a series of malaria control policies and strategies. These included: (1) distribution of ITNs (by social marketing, free to high risk populations, and finally via universal national campaign); (2) IPTp (beginning in 2003); (3) use of ACT (launched in 2006) and a test-and-treat policy (implemented in 2010); (4) IRS (launched in 2008 in 2 districts). The greatest investment came after 2006 from sources including GOM, the Global Fund to Fight AIDS, Tuberculosis and Malaria, and PMI

In Mali, malaria continues to be a public health problem of major significance, representing the primary cause of morbidity, mortality and absenteeism at work and school [8]. In 2012, Mali recorded 2.2 million malaria cases in health facilities, accounting for 42% of all outpatient visits for all age groups. A total of 1900 fatal malaria cases were reported by the Ministry of Health [9]. *Plasmodium falciparum* is responsible for more than 90% of these malaria infections. The primary vectors responsible for malaria transmission include *Anopheles gambiae* sensu stricto (in the rainy season from June to October), *Anopheles funestus* (cold dry season from December to January), *and Anopheles arabiensis* (hot dry season from March to May) [10, 11]. Between 2001 and 2012, the Government of Mali (GOM) and its international development partners invested heavily (more than US$600 million) in a series of malaria control interventions (Fig. 1). These included: (1) distribution of insecticide-treated nets (ITNs) (by social marketing, free to high-risk populations, and via universal national campaign); (2) intermittent preventive treatment in pregnancy (IPTp) (beginning in 2003); (3) use of artemisinin combination therapy (ACT) (launched in 2006) and a test-and-treat policy (implemented in 2010); (4) indoor residual spraying (IRS) (launched in 2008 in 2 districts only). The greatest investment came after 2006 from sources including GOM, the Global Fund to Fight AIDS, Tuberculosis and Malaria, and the US President’s Malaria Initiative (PMI).

Despite these substantial national and international investments, no formal evaluation on a national scale of the public health impact of those interventions has been conducted. To address this gap, this report presents a synthesis of the impact of the expansion of malaria control interventions on all-cause mortality of children under 5 years old (ACCM) from 2000 to 2012.

## Methods

### Evaluation design

**Fig. 2:**
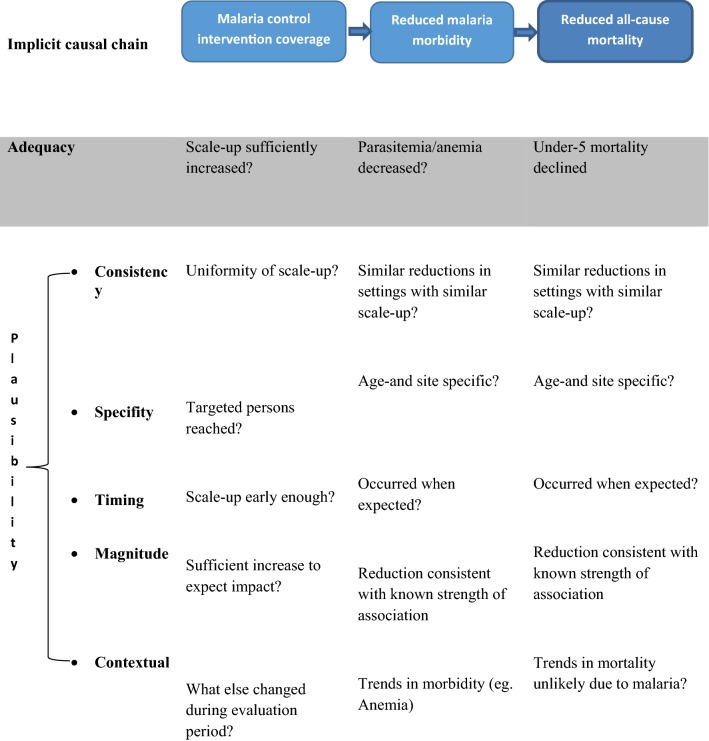
Conceptual framework for an adequacy and plausibility assessment supporting Mali malaria impact evaluation [5]. This evaluation is based on the premise that in high-burden countries, malaria constitutes a sizeable percentage of child mortality, such that improvements in the coverage of malaria control interventions (ITN, IRS, IPTp, case management) should result in a subsequent decline in ACCM. This ‘plausibility argument’ is the current standard for measuring the impact of scale up of malaria control over the past decade. Using ACCM as the primary outcome indicator ensures a robust measure that encompasses both direct and indirect malaria-related mortality. As the association between malaria control interventions and ACCM is mediated by malaria-specific outcomes, this evaluation also includes analyses of several measures of malaria-associated morbidity. Malaria infections and severe anaemia are both outcomes on the causal pathway between malaria control intervention coverage and ACCM. Available morbidity data include prevalence of severe anaemia and of malaria parasitaemia in children. Trends in potential contextual factors influencing the changes in ACCM were also explored

This evaluation is based on the premise that in high-burden countries such as Mali, malaria constitutes a sizeable percentage of child mortality, such that improvements in the coverage of malaria control interventions (ITN, IRS, IPTp, case management) should result in a subsequent decline in ACCM (Fig. 2). This ‘plausibility argument’, as suggested by Rowe et al. [12, 13], and subsequently adopted by the Roll Back Malaria (RBM) Monitoring and Evaluation Reference Group (MERG), is the current standard for measuring the impact of the scale up of malaria control over the past decade. Using ACCM as the primary outcome indicator ensures a robust measure that encompasses both direct and indirect malaria-related mortality. As the association between malaria control interventions and ACCM is mediated by malaria-specific outcomes, this evaluation also includes analyses of several measures of malaria-associated morbidity. Malaria infections and severe anaemia are both outcomes on the causal pathway between malaria control intervention coverage and ACCM. Available morbidity data include prevalence of severe anaemia (< 8 g/dl) and of malaria parasitaemia in children 6–59 months old. Challenges in using morbidity data for the evaluation include the lack of baseline parasitaemia data and the multiple etiologies of anaemia, rendering it a non-specific measure of malaria. Trends in potential contextual factors influencing the changes in ACCM were also explored. More details about the choice of this evaluation design have been described elsewhere [4, 6, 14].

### Data sources and indictors

Estimates of malaria parasitaemia prevalence, anaemia prevalence, ACCM, distribution of demographic characteristics, coverage of malaria interventions, and coverage of other health interventions were obtained mainly from five national, population-based, household surveys conducted in 2001, 2006, 2010, 2012/13, and 2015 [15–19]. As the 2015 survey does not contain data elements needed to calculate ACCM, the 2012/13 survey serves as the endline for the mortality evaluation. Data from the 2015 survey were used to illustrate morbidity trends beyond the primary evaluation period, in part to show the declining burden after the 2012 political crisis. These data were supplemented by routinely collected health data from the *Système Local d’Information Sanitaire* (SLIS), small-area studies and data from other surveys, such as post-net campaign surveys [9, 20–26]. As the northern regions of Mali were excluded from the 2012 Demographic and Health Survey (DHS) and the 2015 Malaria Indicator Survey (MIS) due to security issues, all survey data used in this evaluation have been similarly restricted for comparability. Standard RBM indicators were used to measure malaria intervention coverage [27]. These include the proportion of households that own at least one ITN; the proportion of pregnant women, of children under 5 years of age and of the total population who slept under an ITN the night before the survey; the proportion of women with a live birth in the 2 years preceding the survey receiving at least two doses of sulfadoxine-pyrimethamine (SP) for the prevention of malaria during their most recent pregnancy (IPTp2); the proportion of children under 5 with a fever in the 2 weeks preceding the survey; and, the proportion of children under 5 who received treatment with a first-line anti-malarial among those receiving any anti-malarial medication. Survey data were also used to assess trends in the prevalence of several biomarkers related to malaria. Capillary blood specimens were obtained via finger or heel sticks from children 6–59 months of age who slept in each household the night before the survey. Severe anaemia was defined as haemoglobin < 8 g/dl as measured by HemoCue^®^ instrument. For the detection of *P. falciparum* infection, histidine-rich protein 2-based rapid diagnostic test (RDT) and blood smear were used in the 2010 MIS, the 2012 DHS and the 2015 MIS; however, for this evaluation only blood smear results were used. DHS surveys provided data on contextual factors such as measures of socio-economic status, maternal and reproductive health indicators, nutrition indicators, immunization coverage, and prevalence of other morbidities. Other sources were used for trends in rainfall (University of Columbia [28]) and in gross domestic product. Ethical approval for all surveys was obtained from both ethical committees of University of Sciences, Techniques, and Technologies of Bamako, Mali and from Inter-City Fund (ICF)’s (https://www.icf.com/) institutional review board. Informed consent was signed by each survey interviewer, and analysis was performed on datasets that were anonymized.

### Analyses

#### Temporal trends in malaria control interventions, morbidity and mortality

National trends in intervention coverage, in malaria-associated outcomes (parasitaemia, severe anaemia) and in contextual factors were calculated with corresponding 95% confidence intervals (CIs) for each survey using appropriate adjustments for the two-stage cluster survey design (svy commands in Stata). Intervention coverage was measured for the appropriate population for each indicator (general population, children under 5 years, and pregnant or recently pregnant women age 15–49 years). Malaria infection and severe anaemia were estimated among children of 6–59 months old. Changes in intervention coverage, parasitaemia and severe anaemia were assessed using a Chi square test for linear trend. If 2001 values were not available, 2006 values were used as the baseline. Malaria parasitaemia data were only collected starting in 2010 but continued through 2015, providing a slightly offset timeline for measurement of morbidity trends as compared to the mortality measurements.

Similar stratification was done for mortality rates, which were calculated using data from birth histories of interviewed women and a standard synthetic cohort life table approach to estimate all-cause mortality for children under-five. Each estimate combines data for the 5 years preceding each survey and represents a retrospective 5-year mortality estimate. These estimates were compared for several surveys to reflect temporal trends from pre-baseline through endpoint. Deaths attributable to malaria were also estimated from available routinely collected health facility data [9, 20].

In order to test the hypothesis that expanded coverage in malaria control interventions led to declines in mortality, the timing and magnitude of these trends were examined more closely. Using Hill’s specificity criteria (Fig. 2), the consistency and specificity of the hypothesis were assessed. ACCM was stratified by characteristics including residence (urban/rural), children’s age (6–23 and 24–59 months), malaria risk zone (moderate, 5–40% *Plasmodium falciparum* prevalence (*Pf*PR)_2–10_ [abbrev?] versus high, > 40% *Pf*PR_2–10_) based on data from the Malaria Atlas Project (MAP) [29] and recent evaluation of the malaria risk [10].

#### Temporal trends by age

Both malaria mortality and morbidity vary significantly by age; younger children (< 24 months) are more at risk for both outcomes in high *P. falciparum* transmission areas [30, 31]. ACCM was therefore stratified by age categories (6–23 and 24–59 months) and trends were compared.

#### Temporal trends by malaria endemicity

To assess if greater impact was observed in higher endemicity areas, ACCM was stratified by level of malaria endemicity (moderate, 5–40% *Pf*PR_2–10_ versus high, > 40% *Pf*PR_2–10_) using data from MAP and the new epidemiological profile of malaria in Mali [10, 29].

#### Accounting for contextual factors

Trends in annual precipitation and temperature over time were analysed to assess potential impact on malaria transmission. Mali has seasonal variation in rainfall and temperatures: the rainy season (corresponding to the malaria transmission period) is from June to September, the cold dry season from October to February and the hot dry season from March to May; the annual mean temperature ranges from 12 to 34 °C. A Weighted Anomaly of Standardized Precipitation (WASP) Tool [28] was used to estimate the change in rainfall over the evaluation period relative to baseline (2001–2006). Temperature and WASP data were combined to assess over time if the prevailing climate in Mali was more or less suitable for malaria transmission relative to baseline. Changes in other contextual factors between baseline (2001) and endpoint (2012–2013) were estimated with 95% CIs and per cent changes (relative). A Z-test using the standard errors in the Z-score formula was used to assess differences in baseline and endpoint values.

#### Plausibility assessment

The consistency, specificity, timing, and magnitude of changes in intervention coverage and in impact indicators were assessed considering potential impact of other contextual factors likely to influence child survival. Data on malaria-specific outcomes were also assessed for potential modifying effects on the associations between malaria control intervention coverage and ACCM for the time periods for which they were available.

## Results

### Coverage of interventions

Coverage of malaria control interventions improved significantly from 2006 to 2012 (Table 1). Household ownership of ITNs increased from 49% of households in 2006 to 84% in 2012 and 93% of households by 2015. In line with this expansion of ownership, use of ITNs among high-risk populations also increased over the same period. Among children under five, ITN use increased from 26% in 2006 to 69% in 2012 and 71% by 2015. Similarly, ITN use among currently pregnant women increased to 78% in 2015 and 73% in 2012, from 28% in the 2006 survey. Pregnant women in Mali also receive IPTp with SP to prevent malaria in pregnancy. Over the time period of the study, coverage of at least 2 doses of IPTp during a woman’s most recent pregnancy increased from 10% in 2006 to 29% in 2012 and 38% in 2015. In terms of treatment for malaria, the drug policy evolved over the time period. In 2001, when parasitological diagnosis was not routine and chloroquine was the first-line drug, 92% of children under five with a fever received treatment with chloroquine plus other complementary drugs. By 2005, the national policy had changed to artesunate-amodiaquine (ASAQ) as the first-line treatment, which further evolved to both ASAQ and artemether-lumefantrine (AL) by 2010. In 2010, 23% of children under five with a fever who received an anti-malarial received ACT as first-line treatment. This percentage decreased to 19% in 2012 (p = 0.32) but increased to 29% by 2015, although the difference is not statistically significant (p = 0.09).

**Table 1:** Trends of malaria intervention coverage indicators, Mali, 2000–2015

Interventions	2001 % (95% CI) N	2006 % (95% CI) N	2010 % (95% CI) N	2012–2013 % (95% CI) N	2015 % (95% CI) N	Change^a^	p^£^
Nets							
Household ownership	n/a	40.5 (37.3–42.7) 31,910	62.9 (60.3–65.4) 7883	70.6 (69.4–71.8) 47,829	n/a	30.1	< 0.0001
ITNs							
Household ownership ≥1	n/a	49.4 (46.6–52.2) 11,109	87.2 (84.1–89.7) 1428	84.4 (83.1–85.6) 10,105	93.0 (91.8–94.1) 4240	43.6	< 0.0001
Use (children < 5 yrs)	n/a	26.4 (24.0–29.0) 11,640	72.6 (68.2–76.7) 1801	69.0 (67.2–70.7) 10,634	71.2 (68.9–69.6) 7880	44.8	< 0.0001
Use (pregnant women)	n/a	27.6 (23.4–32.2) 1677		73.2 (69.6–76.5) 1200	77.9 (74.4–81.0) 774	50.3	< 0.0001
Use (all persons)	n/a	20.7 (18.9–22.5) 61,758	56.9 (53.8–59.9) 8721	60.5 (59.0–62.0) 55,836	63.9 (62.0–65.8) 37,755	43.2	< 0.001
IPTp during last pregnancy							
2+ doses	n/a	10.1 (8.6–12.0) 4989	NA	28.5 (26.3–30.9) 3965	37.8 (34.5–41.1) 3017	27.7	< 0.0001
Recommended first-line malaria treatment							
	92.4* (90.0–94.3) 1049	5.5^b^ (3.6–8.3) 677	23.3^c^ (15.8–32.9) 241	19.0^c^ (13.4–26.3) 187	28.9^c^ (23.0–35.8) 590	5.6	0.0171

* Chloroquine used as first-line therapy in 2001

^£^ *P* value of Chi squared for the two time points indicating the changes above

^a^Absolute change from 2006 to 2015, except for recommended first-line malaria treatment where 2010 was the starting point

^b^Sulfadoxine-pyrimethamine used in 2006

^c^Artemisinin combination therapy used in 2010, 2012–2013, and 2015

### All-cause child mortality

**Fig. 3:**
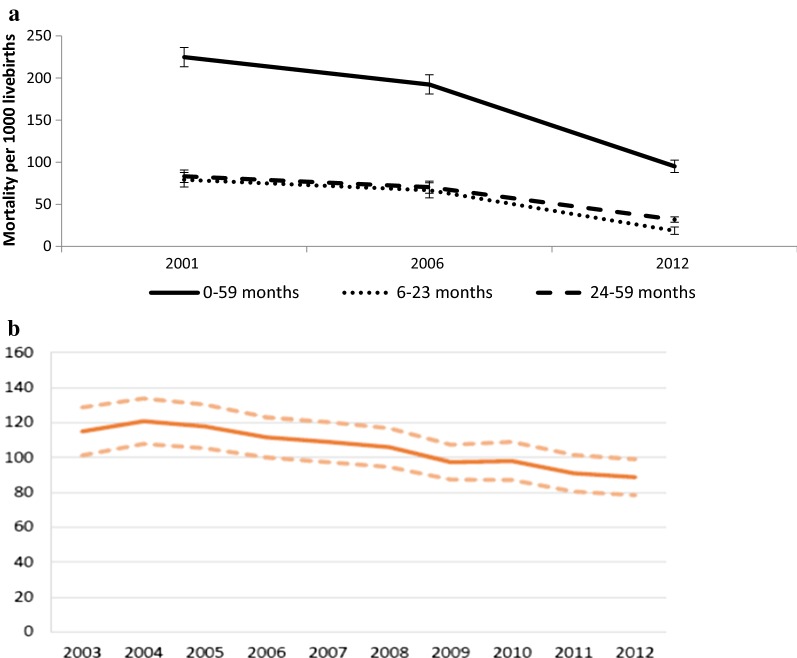
Mortality per 1000 live births from Demographic Health Surveys and routine data, Mali. **a** Mortality rates from DHSs (2001, 2006, 2012–2013); **b** all-cause mortality according to routine data from health information system from 2003 to 2012

Under-five, all-cause mortality rates per 1000 live births declined from 225 in 1997–2001 to 192 in 2002–2006 and 95 in 2008–2012. A similar trend was also observed in children 6–23 and 24–59 months (Table 4, Fig. 3a). Routine data also showed a decline in all-cause under-five mortality rates per 1000 live births from 2003 to 2012 (Fig. 3b).

### Parasitaemia and anaemia

Representative estimates of malaria parasitaemia prevalence are only available from two surveys during the evaluation period: the 2010 Anaemia and Parasitaemia Survey and the 2012 DHS. These surveys are at the end of the evaluation period and are only 2 years apart and therefore offer evidence by which to evaluation the effect of malaria control interventions on ACCM. A subsequent survey, the 2015 MIS, also collected data on these outcomes. The trends in these outcomes over the 2010–2015 period are shown here for illustrative purposes although it is difficult to directly link these findings with the plausibility argument. There was a significant increase in the prevalence of *P. falciparum* infection among children 6–59 months of age from 2010 to 2012 (p < 0.0001) (Table 2), although this was followed by a decrease in 2015 survey, which was significant compared to the 2010 baseline (p < 0.036). The decline in the rural areas, where transmission is higher, was more pronounced than the decline in urban areas. In contrast, the prevalence of severe anaemia which decreased significantly from 2010 (26.3%) to 2012 (20.6%) (p < 0.0001) continued to decline in the 2015 (19.9%) (Table 3).

**Table 2:** Prevalence of *Plasmodium falciparum* carriage among children under 5 years old in Mali, 2010–2015

Characteristics	2010 % (95% CI) N	2012–2013 % (95% CI) N	2015 % (95% CI) N	Change^a^	p-value^≠^	Change^b^	p-value^≠≠^
Overall							
	38.6 (32.6–45.0) 1583	51.6 (48.5–54.8) 4699	35.8 (31.7–40.2) 7071	13	< 0.0001	− 2.7	0.0360
Age categories (years)							
6–11	15.9 (10.0–24.2) 159	41.4 (36.0–47.1) 492	23.6 (18.4–29.8) 784	25.5	< 0.0001	7.7	0.0295
12–23	31.0 (24.5–38.5) 351	46.8 (42.7–50.9) 978	28.5 (24.5–33.0) 1477	15.8	< 0.0001	− 2.5	0.3438
24–35	42.4 (34.1–51.1) 383	50.9 (46.5–55.2) 1030	37.9 (33.2–42.8) 1511	8.5	0.0041	− 4.5	0.1165
36–47	43.8 (35.5–52.5) 348	55.0 (50.6–59.2) 1147	39.0 (34.0–44.2) 1629	11.2	0.0002	− 4.8	0.1041
48–59	47.2 (38.9–55.7) 342	58.0 (53.7–62.1) 1052	43.0 (37.2–48.9) 1681	10.8	0.0004	− 4.2	0.1670
Place of residence							
Urban	4.5 (1.9–10.4) 301	16.8 (13.5–20.6) 863	13.1 (9.5–17.7) 1349	12.3	< 0.0001	8.6	< 0.0001
Rural	46.6 (39.7–53.6) 1282	59.5 (55.9–62.9) 3836	41.2 (36.3–46.2) 5733	12.9	< 0.0001	− 5.4	< 0.0001
Regions							
Kayes	27.1 (15.0–43.9) 207	36.9 (29.7–44.7) 611	27.4 (20.3–35.9) 1083	9.8	0.0110	0.3	0.9127
Koulikoro	40.4 (27.7–54.4) 325	50.2 (41.7–58.8) 1028	34.8 (25.9–44.8) 1481	9.8	0.0018	− 5.6	0.0594
Sikasso	58.5 (47.1–69.1) 230	62.1 (54.9–68.9) 1141	41.6 (34.6–48.9) 1321	3.6	0.3275	− 16.9	< 0.0001
Segou	41.6 (27.0–57.9) 409	55.7 (49.9–61.3) 906	36.7 (28.2–46.1) 1300	14.1	0.0002	− 4.9	0.0764
Mopti^c^	50.2 (36.7–63.7) 229	70.6 (65.4–75.4) 610	59.8 (46.8–71.5) 1087	20.4	< 0.0001	9.6	0.0075
Bamako	2.1 (0.3–14.4) 183	9.9 (7.4–13.0) 403	6.0 (3.9–9.1) 809	7.8	0.0010	3.9	0.0354

^a^Percent absolute change from 2010 to 2012

^b^Percent absolute change from 2010 to 2015

^c^Study was conducted in selected areas of the region of Mopti which were retained for the overall analysis

^≠^p-value of Chi squared comparing proportions of 2010 and 2012

^≠≠^p-value of Chi squared for linear trend in proportions across the three surveys

**Table 3:** Prevalence of severe anaemia among children under 5 years old in Mali, 2010–2015

Characteristics	2006 % (95% CI) N	2010 % (95% CI) N	2012–2013 % (95% CI) N	2015 % (95% CI) N	Change^a^	p-value^≠^
Overall	22.5 (20.0–25.1) 3239	26.3 (22.5–30.5) 1580	20.6 (18.9–22.4) 4745	19.9 (17.4–22.6) 7081	− 2.6	< 0.0001
Age categories (years)						
6–23	31.8 (28.2–35.6) 1099	33.0 (27.4–39.1) 508	25.2 (22.6–28.0) 1485	23.1 (20.5–26.0) 2261	− 8.7	< 0.0001
24–59	17.7 (15.3–20.4) 2139	23.1 (19.3–27.5) 1071	18.5 (16.5–20.6) 3259	18.4 (15.7–21.3) 4821	0.7	0.0018
Endemicity						
Moderate	21.2 (15.6–28.1) 515	15.1 (15.6–28.1) 515	12.5 (9.5–16.2) 689	11.6 (9.3–14.4) 2015	− 9.6	< 0.0001
High	22.9 (10.1–25.8) 2700	28.3 (24.0–33.1) 1325	22.0 (20.1–24.1) 4000	23.2 (20.1–26.5) 5066	0.3	0.00004
Place of residence						
Urban	11.1 (8.1–15.0) 875	9.7 (7.1–13.1) 300	8.4 (6.7–10.6) 879	7.7 (6.4–9.3) 1349	− 3.4	0.0470
Rural	26.7 (24.1–29.5) 2363	30.2 (25.8–35.0) 1279	23.3 (21.4–25.5) 3866	22.7 (19.9–25.9) 5733	− 4.0	< 0.0001
Regions						
Kayes	22.6 (16.1–30.9) 512	31.8 (22.4–43.0) 206	18.9 (15.4–22.9) 613	19.9 (15.6–24.4) 1083	− 3.4	0.0004
Koulikoro	25.1 (20.7–30.1) 659	21.3 (14.7–29.8) 322	20.4 (14.4–25.1) 1038	19.8 (15.8–24.6) 1481	− 4.0	0.0464
Sikasso	27.0 (22.2–32.3) 681	34.6 (27.9–42.0) 230	21.1 (17.6–25.0) 1157	19.4 (15.7–23.7) 1321	− 2.7	< 0.0001
Segou	25.6 (21.4–30.3) 618	28.7 (19.3–40.3) 409	20.5 (16.7–25.0) 909	20.3 (16.7–24.4) 1300	− 5.3	0.0005
Mopti^b^	15.8 (8.3–28.1) 382	31.0 (22.4–41.1) 229	30.1 (25.1–35.6) 611	29.5 (19.6–41.8) 1087	13.7	< 0.0001
Bamako	11.5 (8.4–15.6) 387	7.4 (4.9–11.1) 183	8.3 (6.2–11.0) 417	7.4 (5.9–9.4) 809	− 4.1	0.1021

^a^Percent absolute change from the first survey to the last survey

^b^Study was conducted in selected areas of the region of Mopti which were retained for the overall analysis

^≠^p-value of Chi squared for linear trend in proportions across the surveys

### Plausibility argument 1: temporal associations between intervention coverage and ACCM

**Fig. 4:**
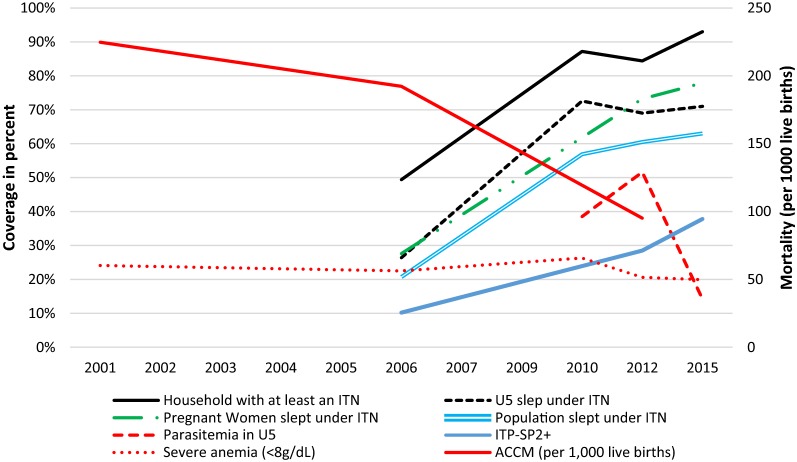
Trend of malaria interventions coverage, malaria morbidity (2001–2015), and all-cause mortality in under five (2001–2012), Mali. Declines seen in ACCM were consistent with the timeframe of scale up of malaria control interventions. There is a steady decline in child mortality from 1997 to 2001 onwards, which was more substantial between 2002–2006 and 2008–2012 than in the first half of the evaluation period. The more recent time period corresponds to the period in which malaria interventions (ITNs were distributed nationally and first-line malaria treatment changed to ACT) were scaled up

The declines seen in ACCM were consistent with the timeframe of the scale up of malaria control interventions (Fig. 4). While Mali saw steady declines in child mortality from the period 1997–2001 onwards, the declines were more substantial in the period between 2002–2006 and 2008–2012 (from 192 per 1000 live births to 95 per 1000 live births, a decline of 51%) than in the first half of the evaluation period (from 225 per 1000 live births to 192 per 1000 live births, a decline of 14%). The more recent time period corresponds to the period in which ITNs were distributed nationally and first-line malaria treatment had changed to ACT.

### Plausibility argument 2: age groups and ACCM

If a large portion of the deaths among children under five is due to malaria, then declines in mortality should be greater among younger children, who are at a greater risk of death from the disease. Among children 6–23 months, mortality declined from 79 deaths per 1000 to 19 deaths per 1000, a 76% reduction between 1997–2001 and 2008–2012. By comparison, mortality among those 24–59 months experienced a 61% drop from 83 to 32 deaths per 1000 (Table 4, Fig. 3a).

**Table 4:** Trends in all-cause mortality (per 1000 live births) among 0–59 months of age in Mali, 2001, 2006, and 2012, by background characteristics

Factors	2001 % (95% CI)	2006 % (95% CI)	2012 % (95% CI)	Change^a^	Change^b^
Age in months					
0–59	224.8 (213.3–236.2)	192.3 (180.8–203.6)	95.1 (87.8–102.3)	− 14.5	− 50.5
6–23	79.1 (71.6–86.5)	66.6 (59.3–73.9)	18.6 (15.4–21.8)	− 15.8	− 72.1
24–59	83.3 (74.7–91.8)	70.4 (61.4–79.4)	31.9 27.6–36.2)	− 15.5	− 54.7
Gender					
Male	230. 2 (214.8–245.4)	192.8 (179.2–206.1)	108.5 (98.5–118.4)	− 16.2	− 43.7
Female	219.3 (204.4–233.9)	191.8 (173.5–209.7)	80.8 (71.6–89.9)	− 12.5	− 57.9
Residence					
Urban	179.9 (156.2–202.9)	132.8 (105.8–159.1)	59.2 (47.6–70.6)	− 26.2	− 55.4
Rural	238.2 (225.1–251.2)	213.4 (201.4–225.2)	103.2 (94.6–111.7)	− 10.4	− 51.6
Endemicity					
Moderate	NA	130.7 (110.1–150.8)	60.3 (46.4–74.0)	NA	− 53.9
High	NA	204.5 (191.5–217.4)	101.7 (93.4–110.0)	NA	− 50.3
Regions					
Kayes	241.3 (216.5–265.2)	164.9 (138.4–190.6)	92.8 (75.3–109.9)	− 31.7	− 43.7
Koulikoro	218.0 (193.5–241.8)	210.8 (187.1–233.8)	86.5 (70.0–102.8)	− 3.3	− 59.0
Sikasso	223.5 (195.4–250.6)	213.0 (194.4–231.2)	109.9 (91.3–128.1)	− 4.7	− 48.4
Segou	250.6 (221.2–278.9)	228.6 (203.9–252.7)	102.2 (85.2–119.0)	− 8.8	− 55.3
Mopti (limited areas)	260.6 (227.7–292.4)	189.3 (140.1–235.7)	102.8 (81.9–123.2)	− 27.4	− 45.7
Bamako	129.8 (109.1–150.0)	99.0 (81.1–116.6)	54.6 (40.8–68.2)	− 23.7	− 44.8

*NA* non-applicable because of absence of data

^a^Relative change from 2001 to 2006

^b^Relative change from 2006 to 2012

### Plausibility argument 3: residence and ACCM

If a significant portion of child mortality is due to malaria, it stands to reason that a greater decrease in the proportion of deaths would be seen among children living in rural areas of high transmission, and thus at higher risk of malaria death, compared to those of urban areas where transmission is moderate. While mortality rates declined in both urban and rural areas of Mali, the decrease in ACCM was greater for children living in rural areas (from 238 deaths per 1000 live births in 1997–2001 to 103 deaths per 1000 births in 2008–2012) compared to urban areas (180 deaths per 1000 live births in 1997–2001 to 59 deaths per 1000 live births in 2008–2012).

### Plausibility argument 4: contextual factors

#### Climate

A warmer, wetter climate is associated with increased malaria transmission due to high populations of the vector. Climate was measured using the WASP index, which varied significantly over the evaluation period in Mali. There was a significant increase in the rainfall during the period of 2006–2012 compared to that of 1990–2005. A similar trend was also observed in the minimum temperature which was higher in the period of 2006–2012 compared to that of 2000–2005. These conditions are typically favourable for malaria transmission and may have contributed to the higher parasitaemia seen in the later period. These conditions might be expected to contribute to increased mortality and therefore do not explain the reductions in ACCM observed during the evaluation period.

#### Socio-economic and political factors

The per capita gross domestic product (GDP) increased from $0.7 million in 2003 to $23.5 million in 2012, the health budget also increased from $1 million in 2007 to $2.5 million in 2012 with a peak of $9 million in 2009. There was a significant increase in the proportion of households with improved water source (43.0% in 2001 and 66.0% in 2012) and improved toilets (6.7% in 2001 and 17.9% on 2012). Similar trends were observed in the proportion of households with modern floors and with electricity (Table 5). Improvements in socio-economic factors could contribute to the observed reductions in ACCM. However, the political upheaval and insecurity in 2012 might be expected to contribute to increased parasitaemia prevalence in 2012 and mortality, and therefore does not explain the reductions in ACCM observed during the evaluation period.

**Table 5:** Prevalence of contextual factors influencing all-cause mortality over time in Mali, 2001 versus 2012–2013

Factors	2001 % (95% CI) N	2006 % (95% CI) N	2012–2013 % (95% CI) N	Change^a^	p^£^
Socio-economic					
Household with improved water source	43,0 (39–47.1) 10,902	57.5 (53.6–61.3) 11,109	66.0 (62.5–69.3) 10,105	23	< 0.0001
Households with improved toilets	6.7 (5.3–8.4) 10,902	10.2 (8.7–11.8) 11,109	17.9 (15.8–20.2) 10,105	11.2	< 0.0001
Household with modern floor material	19.8 (19.9–23.1) 10,902	27.2 (23.5–31.2) 11,109	28.4 (25.7–31.2) 10,105	8.6	< 0.0001
Household with electricity	11.6 (9.6–14.0) 10,902	17.8 (15.3–20.5) 11,109	25.6 (22.9–28.8) 10,105	14	< 0.0001
Maternal and reproductive					
ANC visits 4+	31.0 (28.1–34.0) 7528	63.9 (61.1–66.7) 7944	41.2 (38.7–43.8) 6773	10.2	< 0.0001
Delivery at health centre	24.4 (21.4–27.7) 12,013	47.7 (43.7–51.8) 12,633	55.0 (51.5–58.5) 10,402	30.6	< 0.0001
Delivery by qualified staff	25.5 (22.0–29.3) 12,013	51.7 (47.9–55.5) 12,633	40.1 (36.7–43.5) 10,402	14.6	< 0.0001
Last pregnancy protected against NNT (at least 2 doses of NNT)	32.9 (30.4–35.6) 7528	48.7 (45.7–51.8) 7944	36.6 (34.3–38.8) 6773	3.7	< 0.0001
Vitamin A postnatal supplementation	18.8 (16.7–21.0) 7528	42.2 (39.3–45.2) 7944	50.2 (47.6–52.8) 6773	31.4	< 0.0001
Nutrition in children					
Vitamin A supplementation	41.4 (38.4–44.5) 8815	74.1 (71.4–76.6) 9666	59.8 (57.0–62.4) 8667	18.4	< 0.0001
Stunting	42.2 (40.3–44.0) 10,099	37.6 (35.9–39.4) 10,430	38.3 (36.2–40.4) 4857	− 3.9	< 0.0001
Underweight	28.6 (26.7–30.5) 10,099	26.6 (25.0–27.9) 10,430	25.5 (23.6–27.4) 4857	− 3.1	< 0.001
Emaciation	12.0 (10.8–13.4) 10,099	14.8 (13.9–15.9) 10,430	12.7 (11.1–14.4) 4857	0.7	< 0.0001
Low birth weight	3.3 (2.7–4.0) 12,013	3.9 (3.4–4.6) 12,633	4.7 (4.0–5.5) 10,402	1.4	< 0.0001
Immunization coverage					
BCG	70.4 (67.0–73.6) 2003	78.1 (74.5–81.3) 2325	83.3 (80.8–86.1) 1846	12.9	< 0.0001
DPT3	41.1 (36.9–45.5) 2003	69.2 (65.4–72.7) 2325	63.1 (59.7–66.3) 1846	22.0	< 0.0001
Polio3	41.3 (37.4–45.2) 2003	63.6 (60.1–67.1) 2325	50.0 (46.5–53.6) 1846	8.7	< 0.0001
Measles	50.2 (46.6–53.7) 2003	63.6 (65.1–73.9) 2325	71.7 (68.7–74.5) 1846	21.5	< 0.0001
Total coverage	30.0 (26.5–33.8) 2003	49.6 (45.8–53.5) 2325	38.9 (35.5–42.4) 1846	8.9	< 0.0001
Morbidity (past 2 weeks)					
Prevalence of diarrhoea	17.4 (16.1–18.8) 10,166	13.9 (12.7–15.1) 10,959	8.6 (7.7–9.6) 9655	− 8.8	< 0.0001
Prevalence of ARI	9.6 (8.6–10.7) 10,166	5.9 (5.2–6.8) 10,959	3.2 (2.7–3.8) 9655	− 6.4	< 0.0001

*ANC* antenatal clinic, *NNT* neonatal tetanus toxoid, *BCG* Bacillus Calmette-Guérin, *DPT* diphteria pertusis tetanus, *ARI* acute respiratory infection

^a^Percent absolute change from 2001 to 2012–2013

^£^p-value of Chi squared for linear trend in proportions across the three surveys

#### Maternal and reproductive factors

The proportion of pregnant women attending 4 or more antenatal clinic (ANC) visits increased from 2001 (31.0%) to 2006 (64.0%), followed by a decrease in 2012 (41.2%). A similar trend was observed in the proportion of pregnant women delivered by a qualified provider and the proportion of women who received at least 2 doses of tetanus toxoid. In contrast, the proportion of women who delivered at a health centre and the proportion receiving postnatal vitamin A supplementation continued upward between 2006 and 2012 although increases were small (Table 5). Given the declining coverage in most maternal reproductive health factors and the minimal improvements in other factors during the period of the most significant reduction in ACCM, these factors are unlikely to be important drivers in the observed ACCM decline during the evaluation period.

#### Nutrition

The proportion of vitamin A supplementation in children increased from 41.4% in 2001 to 74.1% in 2006 and decreased to 59.8% in 2012. The proportion of children with low height-for-age and low weight-for-age declined by small but significant amounts (4 and 3.1%, respectively) over the evaluation period. The proportion of children with low weight-for-height did not change significantly over the evaluation period (12.0% in 2001 compared to 12.7% in 2012), although a significant increase (2.8%) was seen in 2006 (14.8%). The proportion of low birth weight newborns did not decline over the evaluation period but rather increased from 3.3% in 2001 to 3.9% in 2006 and 4.7% in 2012 (Table 5). Given the minimal reductions or increases in nutrition-associated morbidities over the evaluation period, these factors are unlikely to explain the observed reductions in ACCM.

#### Vaccination

There was an increase in the percentage of children fully vaccinated from 30.0% in 2001 to 49.6% in 2006, followed by a decrease (38.9%) in 2012 (Table 5). These trends are inconsistent with the hypothesis that increase vaccination coverage explains the observed reductions in ACCM.

#### Other morbidity

The prevalence of diarrhoea and acute respiratory infection declined by 8.8 and 6.4%, respectively, over the evaluation period (Table 5). Although reductions in these morbidities may help drive reductions in ACCM, the magnitude of change was relatively small over the evaluation period.

#### Plausibility argument 5: malaria morbidity on the causal pathway

Baseline data on malaria parasitaemia prevalence do not exist in Mali. The first available parasitaemia data are from the 2010 MIS. Between 2010 and 2012, the prevalence of malaria increased from 39 to 52% among children 6–59 months. Overall, from 2010 to 2015 malaria parasitaemia prevalence declined significantly from 38.6 to 35.8% (p = 0.0360). Consistent with the plausibility argument, stratified analyses revealed that the decrease in malaria prevalence was more pronounced in younger children (6–23 months) compared to older children (24–59 months), in rural areas compared to urban areas, and in the four regions where the most significant increase was seen between 2010 and 2012.

Markers of severe anaemia (< 8 g/dl) among children under five remained relatively constant from 2001 through 2010, with a small, but statistically significant decline between 2010 (26.3%) and 2015 (19.9%). However, among children 6–23 months, who are at the highest risk for malaria-related anaemia, there was a statistically significant decline from 2006 (32%) to 2015 (23%), while prevalence among children 24–59 months did not decrease (17.7% in 2006 vs 18.4% in 2015). Declines in severe anaemia were also more pronounced in strata where the greatest declines in malaria prevalence occurred.

## Discussion

Over the period of evaluation, ACCM, used as a standard indicator of malaria programme impact in high endemicity countries of sub-Saharan Africa [27], declined substantially during a period of rapid investment in and expansion of malaria control interventions. The observed mortality trends are consistent with what would be expected if reductions in malaria transmission were an important driver. The temporality of the mortality trends is consistent with the hypothesis that reduction in malaria is a major cause. Although a significant decline in ACCM was observed between 1997–2001 and 2002–2006, a much greater decline was observed between 2002–2006 and 2008–2012, the period corresponding to the rapid expansion of malaria control interventions, including ITN distribution and use, IPTp expansion and improvements in malaria case management. In further support of this hypothesis, the greatest mortality decline was seen in children 6–23 months who are also at the highest risk for malaria-related morbidity and mortality [30]. These findings, derived from household survey data, are also supported by the health facility data reported to the routine information system. These routinely reported data showed a decrease trend in ACCM from 2003 to 2012 among children under five, with higher decrease from 2007 to 2012 (Fig. 3b), a time period after the country officially adopted ACT as first-line treatment for uncomplicated malaria [26]. A similar decline was seen among older children and adults. The total number of deaths in health facilities from all causes for both children under five and for older age groups increased between 2000 and 2010. This may be explained by increases in access to health services, in parasitological confirmation of cases and in reporting of cases during this period. Between 2010 and 2012, when malaria diagnostics were more widely used in Mali (~ 50% of malaria cases confirmed by RDT or microscopy were treated in a health centre), weekly surveillance data indicate a decline in malaria deaths.

Even though the decline in ACCM was concurrent with an increase in the coverage of malaria control interventions between 2001 and 2012, it is important to note that other contextual factors might have contributed to the observed declines in mortality as suggested by previous studies [4, 5, 13, 32, 33]. During the same timeframe, a number of other indicators of socio-economic conditions and coverage of other health interventions also improved. These include GDP, access to potable water and improved sanitation facilities, although their benefit was not equitably distributed across the country (Gini coefficient = 0.033). Several maternal and child health interventions also expanded coverage during this period, including use of antenatal care, delivery with a skilled attendant, and vaccination for preventable childhood diseases. Despite the expanded coverage over the evaluation period as a whole, declines were noted in coverage of many of these interventions between 2006 and 2012 and per cent of population covered with these services remained quite low in general, indicating their small contribution to the observed decrease in ACCM.

Given the significant expanded coverage of malaria control interventions and reduction in ACCM over the last half of the evaluation period, a significant reduction in the prevalence of malaria was expected, as reported by previous authors [5]. Unfortunately, nationally representative data on malaria prevalence are lacking for most of the evaluation period. Data that are available show a significant increase in the prevalence of parasitaemia among children 6–59 months old from 2010 (39%) to 2012 (52%) [17, 18]. Although this increase was observed in all regions, it was most pronounced in the region of Mopti where the prevalence increased from 50% (2010) to 71% (2012). As this increase coincides with a significant improvement in the coverage of malaria major interventions, there is a suggestion of possible failure of malaria interventions in Mali. However, it is important to note that the 12 months or so immediately preceding the endpoint of this evaluation (2012) was a period of political upheaval and insecurity in Mali. The 2012 survey data were collected at a time of large-scale movements of malaria-naïve populations from the north to malaria-endemic regions of the south. The region of Mopti received many displaced people from the north and was shown to be the region the highest prevalence of parasitaemia in 2012. Furthermore, climate conditions could have facilitated an increase in malaria transmission over the period of 2006–2012 and contributed to the temporary disruption of the momentum initiated in malaria control. National rainfall data indicate that the period between 2000 and 2006 was drier than the 15-year average, but the period corresponding to the expansion of malaria control interventions (2006–2012) was significantly wetter than average. Similarly, the annual temperature deviation from the 50-year average was markedly hotter from 2006 to 2012 than for the earlier period. Taken together, these climate variations could have facilitated an increase in malaria transmission over the latter part of the evaluation period. This, in addition to the political upheaval and insecurity in Mali may have contributed to the increase of malaria prevalence observed between 2010 and 2012 [17].

The malaria prevalence estimates from the 2015 MIS [19] show a significant decline from 2012 levels (36% compared to 52%) thereby providing additional evidence that the 2012 malaria prevalence was an anomaly, perhaps due to political and climate factors, and that malaria intervention strategies are contributing to burden reduction.

Severe anaemia (HB < 8 g/dl) is another indicator of malaria-associated morbidity, although nutritional causes, genetic disorders and other parasitic infections can play a part in anaemia prevalence. The study suggested a small but significant decrease (p = 0.0326) in the prevalence of severe anaemia in children 6–59 months from 23% in 2006 to 21% in 2012. This decrease was more pronounced among young children (6–23 months) compared to older children, and among those living in rural compared to urban areas. The decline in the indicated strata was persistent during the expansion period of malaria control interventions from 2006 to 2012, and when data of 2010–2015 only are examined, the decline is more substantial, supporting the plausibility argument that malaria interventions were having an indirect effect in reducing the burden of anemia.

During the evaluation period the health sector expanded, providing increased access to health care. The number of health facilities increased and community case management was expanded, which may have contributed to detection and reporting of a greater number of malaria cases. Towards the end of the evaluation period, RDTs were introduced and diagnostics were improved, and case management was pushed out to the community level through community health workers, also potentially contributing to an increase in reported cases. Weekly reporting of malaria cases was only functional in the epidemic-prone regions of the north until 2008 when it was extended to the entire country. In general, the routine information system benefitted from a number of investments to improve the quality and timeliness of reporting which could have influenced the observed increase in reported cases.

In summary, the results of this evaluation show an important decline in ACCM corresponding to the timeframe of the rapid increase in coverage of malaria control interventions. The timing and specificity of the results are consistent with the hypothesis that improved malaria control contributed to observed mortality declines: intervention coverage was largely scaled up after 2006, corresponding to the period of the most dramatic decline in ACCM (Fig. 4), and declines in ACCM were more pronounced in children with the highest malaria risk (rural, younger children).

Malaria morbidity data, such as population-based parasitaemia prevalence, are limited for the beginning of the evaluation period, leading to a lack of reliable baseline data. Observed increases in these indicators for the period between 2010 and 2012 suggest a continued high burden of malaria in Mali. While national-level parasitaemia prevalence increased from 2010 to 2012, that change was likely driven on the one hand by an extreme increase in Mopti region, the region with large numbers of displaced persons from the low malaria burden areas of the north, moving into high transmission zones of the south, and on the other hand by climate conditions favourable to malaria transmission. Thus, parasitaemia prevalence in 2012 is not an accurate reflection of long-term trends (indeed a subsequent survey in 2015 found 36% prevalence in children under five, as well as substantial declines in every region).

## Conclusions

Taken as a whole, the evidence supports the conclusion that malaria control interventions substantially contributed to the observed decline in ACCM in Mali from 2000 to 2012, even in a context of continued high prevalence of parasitaemia and political instability. As Mali returns to a peaceful political status, the strong foundation of malaria control built over the past 15 years provides a sense of optimism for future gains.

## Abbreviations

ACT: artemisinin-based combination therapy
ACCM: all-cause child mortality
ITN: insecticide-treated net
IPTp: intermittent preventive treatment
GOM: Government of Mali
IRS: indoor residual spray
PMI: US President’s Malaria Initiative
RBM: Roll Back Malaria
MERG: Monitoring and Evaluation Reference Group
DHS: Demographics Health Survey
MIS: Malaria Indicator Survey
SP: sulfadoxine-pyrimethamine
RDT: rapid diagnostic test
ASAQ: artesunate-amodiaquine
AL: artemether-lumefantrine
SLIS: Systeme Local d’Information Sanitaire
MAP: Malaria Atlas Project
GDP: gross domestic product
